# Oxidative stress mediates an increased formation of vascular endothelial growth factor in human hepatocarcinoma cells exposed to erlotinib

**DOI:** 10.18632/oncotarget.19055

**Published:** 2017-07-06

**Authors:** Nataliya Rohr-Udilova, Florian Klinglmüller, Martha Seif, Hubert Hayden, Martin Bilban, Matthias Pinter, Klaus Stolze, Wolfgang Sieghart, Markus Peck-Radosavljevic, Michael Trauner

**Affiliations:** ^1^ Division of Gastroenterology and Hepatology, Department of Internal Medicine III, Medical University of Vienna, A-1090 Vienna, Austria; ^2^ Center for Medical Statistics, Informatics and Intelligent Systems, Medical University of Vienna, A-1090 Vienna, Austria; ^3^ Clinical Institute for Laboratory Medicine, Medical University of Vienna, A-1090 Vienna, Austria; ^4^ Institute of Animal Nutrition and Functional Plant Compounds, Department for Farm Animals and Veterinary Public Health, University of Veterinary Medicine, A-1220 Vienna, Austria; ^5^ Clinic Klagenfurth, Division of Gastroenterology and Hepatology, 9020 Klagenfurt am Wörthersee, Austria

**Keywords:** tyrosine kinase inhibitors, erlotinib, vascular endothelial growth factor, cytochrome P450, hepatocarcinoma cell lines

## Abstract

The tyrosine kinase inhibitor erlotinib targets the receptor of epidermal growth factor (EGFR) involved in development of hepatocellular carcinoma (HCC).

Although inefficient in established HCC, erlotinib has been recently proposed for HCC chemoprevention. Since Cyp3A4 and Cyp1A2 enzymes metabolize erlotinib in the liver, the insights into the mechanisms of erlotinib effects on liver cells with maintained drug metabolizing activity are needed.

We applied erlotinib to both commercially available (SNU398, Huh7) and established in Austria HCC cell lines (HCC-1.2, HCC-3). Cyp3A4 and Cyp1A2, microarray gene expression, cell viability, LDH release, DHFC fluorescence were assessed. VEGF expression was analysed by real-time RT-PCR and ELISA.

Higher cumulative expression of erlotinib metabolizing enzymes was observed in HCC-1.2 and HCC-3 cells. Gene expression microarray analysis showed upregulation of VEGF signalling by erlotinib. VEGF was increased up to 134 ± 14% (*n* = 5, *p* = 0.002) in HCC-1.2, HCC-3 and Huh7 cells. Interventions by Cyp1A2 and Mek2siRNA, MEK inhibitor UO126, diphenylene iodonium, as well as a combination of N-acetylcysteine with selenium all inhibited VEGF upregulation caused by erlotinib.

Thus, erlotinib increases VEGF production by mechanisms involving Cyp1A2, oxidative stress and MEK1/2. VEGF may favour angiogenesis and growth of early HCC tumours limiting the therapeutic and chemopreventive effects of erlotinib.

## INTRODUCTION

Erlotinib is supposed to act mainly through the inhibition of tyrosine kinase domain of epidermal growth factor (EGF) receptor [1]. Since erlotinib has been approved for treatment of non-small cell lung cancer, its efficiency to treat other cancers with deregulated EGFR signalling pathway has been investigated.

Upregulation of EGFR signalling pathway in hepatocellular carcinoma (HCC) is supported by several lines of evidences. In particular, EGF is a member of a predictive gene signature for HCC development in human [2]. In addition, EGF polymorphisms which increase EGF stability also increase the risk of HCC [3]. Finally, hepatic overexpression of EGF promotes hepatocarcinogenesis [4].

Although erlotinib diminished viability of HCC cells *in vitro* [5, 6], the *in vivo* data were quite sobering. We have reported the lack of erlotinib efficacy in an orthotopic HCC rat model [7]. Erlotinib monotherapy showed modest effect also in clinical HCC studies [8, 9]. In addition, erlotinib failed to increase the efficiency of sorafenib in a phase III study in a first line HCC therapy [10]. However, case reports suggest that erlotinib could still be a treatment option for certain patients [11].

More recent, a new field of erlotinib application as antifibrotic and thus as a cancer preventive agent has been proposed [12]. The suggested mechanisms included resolution of experimental liver fibrosis, delay of tumour development [12] as well as the inhibition of IL-1 and IL-6 production from liver-derived macrophages [13].

A clinical study on HCC prevention by erlotinib is currently running (https://clinicaltrials.gov/, study identifier NCT02273362). Therefore, a better understanding of the mechanisms and potential side-effects is of clinical relevance. Since early undiagnosed tumours or premalignant lesions can be already present in cancer-predisposed liver, mechanistic data would help to evaluate potential benefits and drawbacks of HCC prevention by erlotinib.

Because erlotinib is metabolized predominately by cytochrome P450 system, particularly by CYP3A4 [14] and CYP1A2 [15], a proper cellular model is required. However, cytochrome P450 expression is low in the most commercially available HCC cell lines. Since drug metabolism by CYP P450 enzymes may increase intracellular free radical formation, interactions with redox-sensitive members of EGFR pathway can be expected. Here, we investigated the effects of erlotinib in special HCC cell lines with retained activity of hepatic cytochromes.

## RESULTS

### Erlotinib and viability of HCC cells

**Figure 1:**
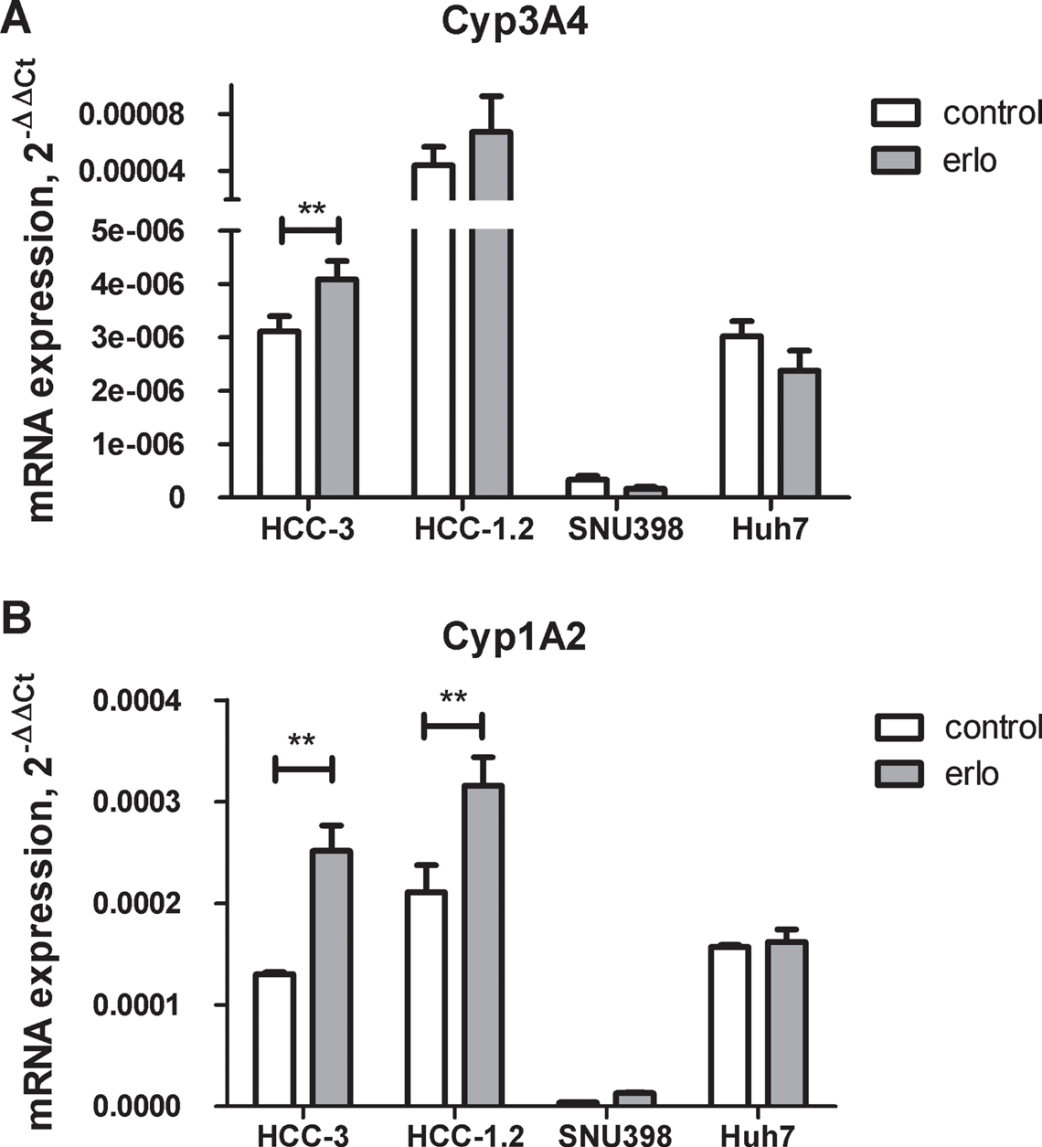
Cytochrome P450 expression in HCC cell lines 10 μM erlotinib (erlo) or solvent control (control) were applied to HCC cells for 3 hours and expression of CYP3A4 (**A**) or CYP1A2 (**B**) was analysed by real-time RT-PCR. ΔΔC_t_ method was applied for quantification using GAPDH as a house keeping gene. The results of *n* = 3 independent experiments are shown. **p < 0.01.

To simulate biological heterogeneity of HCC, four cell lines including Huh7, SNU398 and Austrian hepatocarcinoma cells HCC-1.2 and HCC-3 with maintained activity of hepatic metabolizing enzymes [16] were treated by erlotinib. First, mRNA levels of erlotinib metabolising enzymes Cyp3A4 and Cyp1A2 were compared between the cell lines. As Figure 1A shows, by far the highest mRNA expression of Cyp3A4 was detected in control and erlotinib-treated HCC-1.2 followed by erlotinib-treated HCC-3 cells. The highest Cyp1A2 mRNA expression was detected in erlotinib-treated HCC-1.2 cells followed by erlotinib-treated HCC-3 (Figure 1B). SNU398 cells had the lowest expression of both Cyp3A4 and Cyp1A2. Huh7 expressed Cyp1A2 mRNA at higher levels than Cyp3A4 mRNA and both cytochromes were not further induced by erlotinib in this cell line. The cumulative expression of Cyp3A4 and Cyp1A2 was higher in our established HCC-1.2 and HCC-3 cells compared to commercially available Huh7 and SNU398. Of note, Cyp1A2 exhibited 3 - 88 times higher mRNA levels than Cyp3A4 in all investigated cell lines.

**Figure 2:**
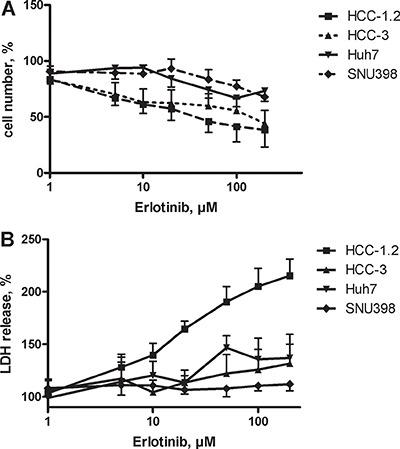
Erlotinib sensitivity of HCC cell lines (**A**) HCC cells were incubated with increasing erlotinib concentrations in a serum free medium containing 1% BSA for 24 h. Neutral red assay was used to quantify viable cells as described in Material and Methods section. (**B**) LDH release.

EGFR, a target of erlotinib, was expressed in all cell lines [17]. As Figure 2A shows, erlotinib diminished the viability of HCC cells in a dose dependent manner. The cells varied in their sensitivity to erlotinib: HCC-1.2 was the most sensitive cell line with LD_50_ = 16.3 ± 4.4 μM, followed by HCC-3 with LD_50_ = 114.3 ± 35.0 μM. Accordingly, LDH release indicative for loss of membrane integrity was increased. The highest LDH release was observed in HCC-1.2 cells indicating necrotic cell death (Figure 2B). Huh7 and SNU 398 were less sensitive to erlotinib (low-sensitive) as compared to HCC-1.2 and HCC-3 (high-sensitive).

Three cell lines HCC-3, Huh7 and SNU398 showed less pronounced LDH release and still some cell loss. Measurements of apoptotic cells revealed that erlotinib did not significantly change apoptosis in HCC-3 but showed a trend towards an increased percentage of early necrotic cells (Supplementary Figure 1A). At our experimental conditions, erlotinib had no impact on apoptosis in Huh7 and SNU398 cells (Supplementary Figure 1B and 1C).

### Microarray analysis of erlotinib effects

To obtain a comprehensive overview on pathways regulated by erlotinib, the most sensitive HCC-1.2 cells were treated with erlotinib and a microarray gene expression analysis was performed. 10 μM erlotinib has been chosen within the C_max_ range measured in human [18]. To reduce the number of potential targets of questionable relevance, we first applied non-specific filtering based on a robust variance measure reducing the probe set. Both, a single gene approach and a pathway linked gene set approach were used. Significantly regulated genes are listed in Supplementary Table 1. Among the common signal transduction pathways, p53 related genes showed the most significant difference due to erlotinib treatment (Supplementary Table 2). In addition, a significant regulation of the map “Development-VEGF-family signalling” (*p* < 0.001) has been identified by a comparative enrichment analysis of the most significantly regulated genes (Supplementary Figure 2). Based on these findings, VEGF expression in erlotinib-treated HCC cells was investigated in greater details.

### Effect of erlotinib on VEGF formation in HCC cells

Real time RT-PCR analysis confirmed the VEGF upregulation by erlotinib identified by microarray analysis in HCC-1.2 cells. In addition, we studied the impact of erlotinib on VEGF in other above erlotinib-sensitive and –insensitive cell lines.

**Figure 3:**
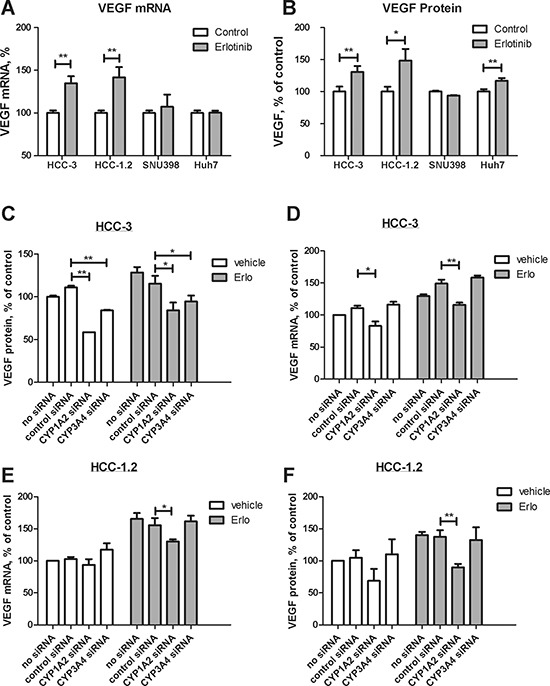
Impact of erlotinib on VEGF formation in HCC cells 10 μM erlotinib were applied to HCC cells for 3 hours (**A**) or for 6 hours (**B**). VEGF expression was determined by real time RT-PCR (A) and by ELISA (B) as described by Experimental Procedures. The results of three independent experiments are shown. HCC-3 cells were transfected by Cyp1A2 and Cyp3A4 siRNA as described in Experimental Procedures and VEGF mRNA (**C**) and VEGF protein (**D**) were investigated under the same conditions as in (A) and (B) respectively. (**E**)–the same as (C) except that HCC-1.2 cells were used. (**F**) – the same as (D) except that HCC-1.2 cells were used. **p < 0.01; *p < 0.05.

High-sensitive HCC-1.2 and HCC-3 cells increased VEGF mRNA and protein levels under erlotinib treatment (Figure 3A and 3B). In low-sensitive Huh7, VEGF increase was observed only at protein level and was much less pronounced than in HCC-1.2 and HCC-3 cells (Figure 3B).

To clarify the contribution of Cyp1A2 and Cyp3A4 to erlotinib-induced VEGF formation, we performed silencing by siRNA. Transfection with respective siRNA decreased mRNA levels of Cyp1A2 and Cyp3A4 up to 70% (Supplementary Figure 3). As Figure 3C–3D show, Cyp1A2 siRNA consistently prevented an induction of both VEGF mRNA and protein by erlotinib in HCC-1.2 and HCC-3 cells. In HCC-3 cells, Cyp3A4 siRNA also decreased VEGF protein, although to a lesser extent than Cyp1A2 siRNA (Figure 3C–3E). These data strongly suggest that Cyp1A2 mainly contributes to VEGF induction by erlotinib in HCC cells.

### Erlotinib and intracellular prooxidants

**Figure 4:**
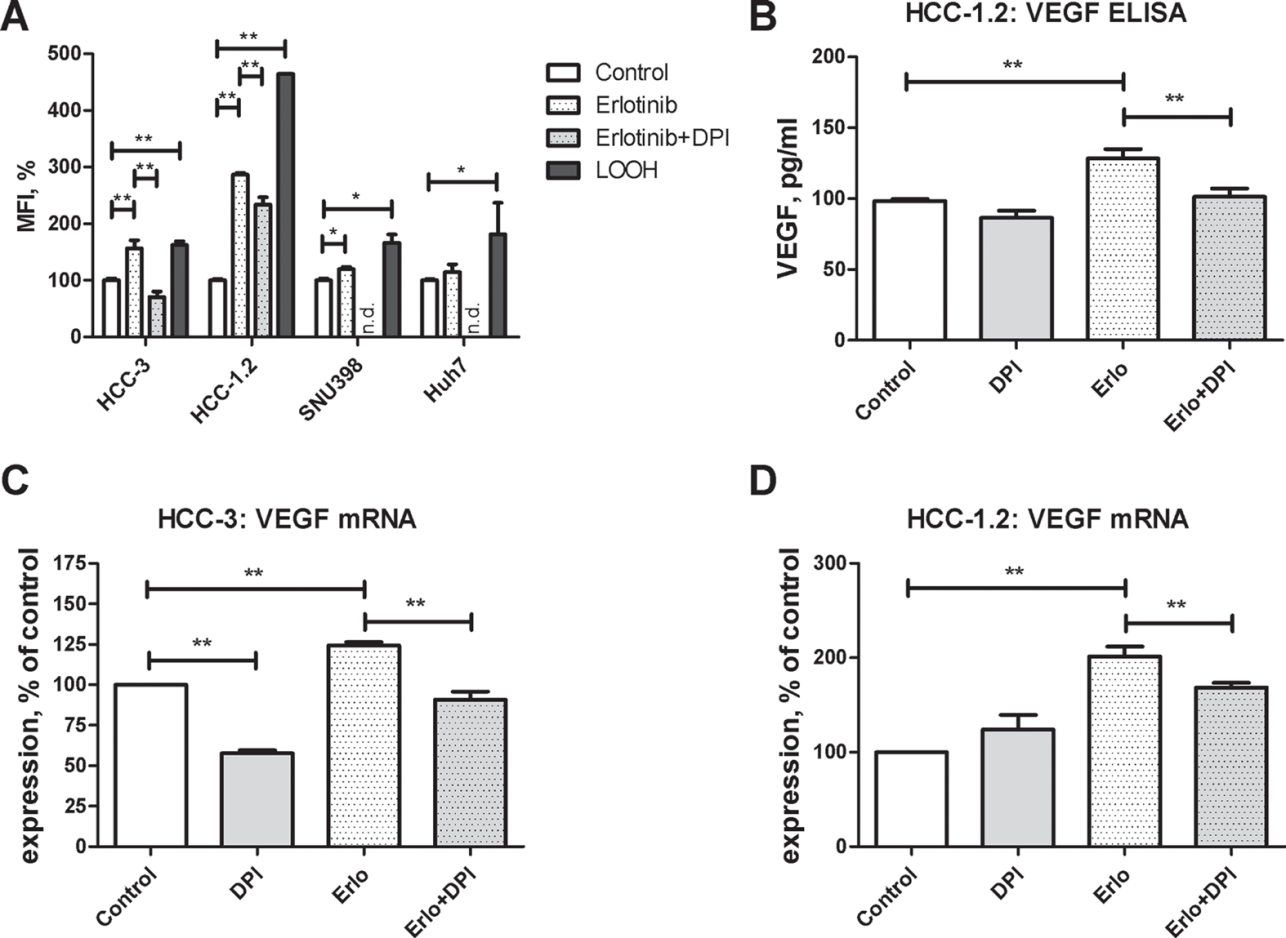
Erlotinib and redox imbalance in cultured HCC cells Cells were incubated for 2 h with 10 μM erlotinib. (**A**) DCFH staining was performed as described in Experimental Procedures section and quantified by FACS analysis. Mean fluorescence intensity (MFI) normalized to control untreated cells was used to reflect redox changes. Following concentrations were applied: 15 μM DPI, 20 μM LOOH, 25 μM DCFH. Impact of DPI on erlotinib-induced VEGF protein (**B**) and mRNA formation in HCC-3 (**C**) and HCC-1.2 (**D**) cells. Cells were treated by 10 μM erlotinib with and without 15 μM DPI for 2 h. Supernatants were collected after the additional 2 h of incubation and VEGF concentration was measured by ELISA. All measurements were performed in duplicates, the results of *n* = 3 independent experiments are shown. n.d. – not determined; *p < 0.05; **p < 0.01.

Since prooxidants can up-regulate VEGF formation in cancer cells [19–21], the impact of erlotinib on prooxidant formation was investigated. For this purpose, redox sensitive DCFH dye which forms a fluorescent DCF product upon two electron oxidation was applied [22]. As a positive control, the cells were treated by a prooxidant linoleic acid hydroperoxide (LOOH) [21]. Erlotinib increased the DCF fluorescence in high-sensitive HCC-3 and HCC-1.2 cell (Figure 4A). In contrast, low-sensitive Huh7 and SNU398 cells showed less pronounced or no fluorescence increase (Figure 4A). Notably, the HCC-1.2 cells with the highest expression of CypP450 enzymes revealed the highest fluorescence induction by erlotinib.

To get additional hints on potential oxidant sources, diphenyleneiodonium (DPI) was applied. Addition of 15 μM DPI for the time of experiment did not affect cellular viability (Supplementary Figure 4). As shown in Figure 4A, DPI inhibited DCF fluorescence in HCC-1.2 and HCC-3 cells. Accordingly, DPI also inhibited VEGF mRNA and protein induced by erlotinib (Figure 4B, 4C). Thus, flavoprotein inhibition by DPI decreases erlotinib-induced VEGF mRNA and protein and flavoproteins are involved in erlotinib-induced prooxidant formation.

### Involvement of MEK1/2

**Figure 5:**
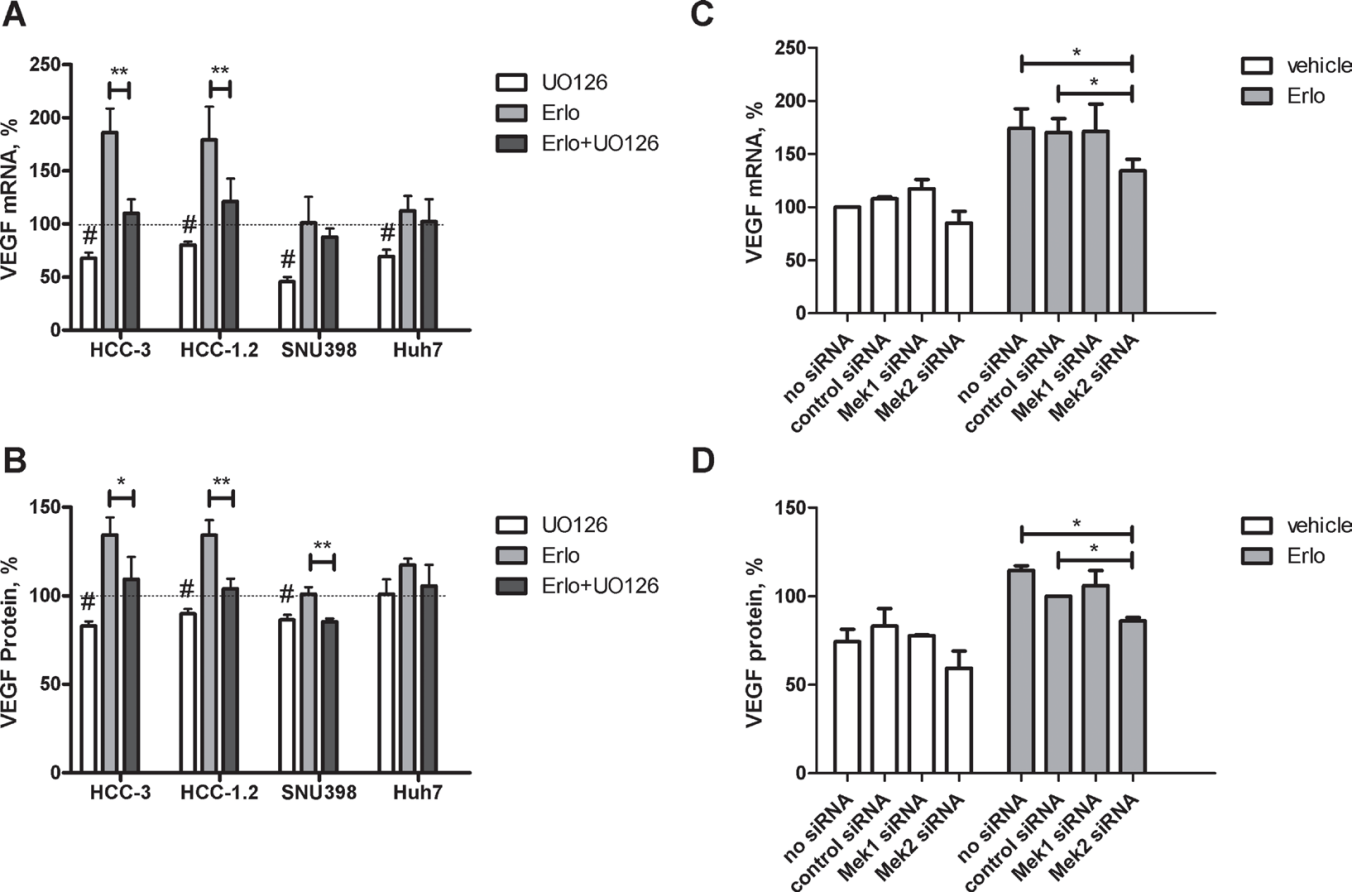
Impact of MEK1/2 on VEGF expression induced by erlotinib in HCC cells HCC-3, HCC-1.2, SNU398 or Huh7 cells were grown in 6 well plates, incubated with 10 μM UO126 or with a solvent for 1 h followed by 10 μM erlotinib for 3 h in a serum free medium. VEGF mRNA was analysed as described in Experimental Procedures (**A**) VEGF protein in supernatants was analysed after additional 3 h in a serum free medium (**B**) The results are presented as % of vehicle-treated controls. *n* = 3, ^#^*p* < 0.01 vs untreated 100% control, *p < 0.05 vs erlotinib; **p < 0.01 vs erlotinib. siRNA against MEK1 and MEK2 was applied to HCC-1.2 cells treated either by vehicle or by 10μM erlotinib. VEGF mRNA was analysed by real-time RT-PCR (**C**) and by ELISA (**D**) according to the same protocol as in Figure 3A–3B.

Since stimulatory signals from EGFR pathway are converged to MEK1/2 [23], impact of MEK1/2 inhibitor U0126 on VEGF stimulation by erlotinib was further investigated. At base line, U0126 decreased VEGF mRNA in all cell lines under investigation (Figure 5A, 5B). U0126 also diminished VEGF protein in HCC-1.2, HCC-3 and SNU398 but not in Huh7 cells. At stimulation by erlotinib, U0126 completely prevented VEGF protein induction in SNU398 cells and partially prevented in HCC-1.2 and HCC-3 cells (Figure 5B). In Huh7, there was only a trend toward VEGF decrease by U0126 without reaching significance.

To further confirm the involvement of MEK1/2 in VEGF upregulation by erlotinib, we performed siRNA studies. For these experiments, HCC-1.2 cell line was selected based on the highest magnitude of VEGF upregulation by erlotinib. To control the efficiency of siRNA inhibition, MEK1 and MEK2 mRNA have been analysed by real-time PCR. As Supplementary Figure 5 shows, siRNAs inhibited the corresponding MEK1 and MEK2 mRNA levels. Even if MEK2 siRNA inhibited MEK1 mRNA levels to some extent, the magnitude of the inhibition (∼30%) was much lower than it could be achieved by MEK1 siRNA (∼95%). Erlotinib did not affect MEK1 and MEK2 mRNA levels (Supplementary Figure 5).

Intervention by MEK2 siRNA - but not by MEK1 siRNA–inhibited erlotinib-induced VEGF formation at both mRNA and protein level (Figure 5C, 5D). Thus, MEK2 is involved in VEGF upregulation by erlotinib in HCC-1.2 cells.

### Combination of N-acetylcysteine and selenium inhibited VEGF formation induced by erlotinib

In order to further explore the hypothesis whether intracellular prooxidants contribute to an increased VEGF formation under erlotinib treatment, the effect of N-acetylcysteine (NAC) and selenium was investigated.

HCC-1.2 and HCC-3 cell lines were chosen for these experiments based on the most pronounced increase of VEGF and DCF fluorescence upon erlotinib treatment. N-acetylcysteine (NAC) and selenium were applied both as single agents as well as in combination.

**Figure 6:**
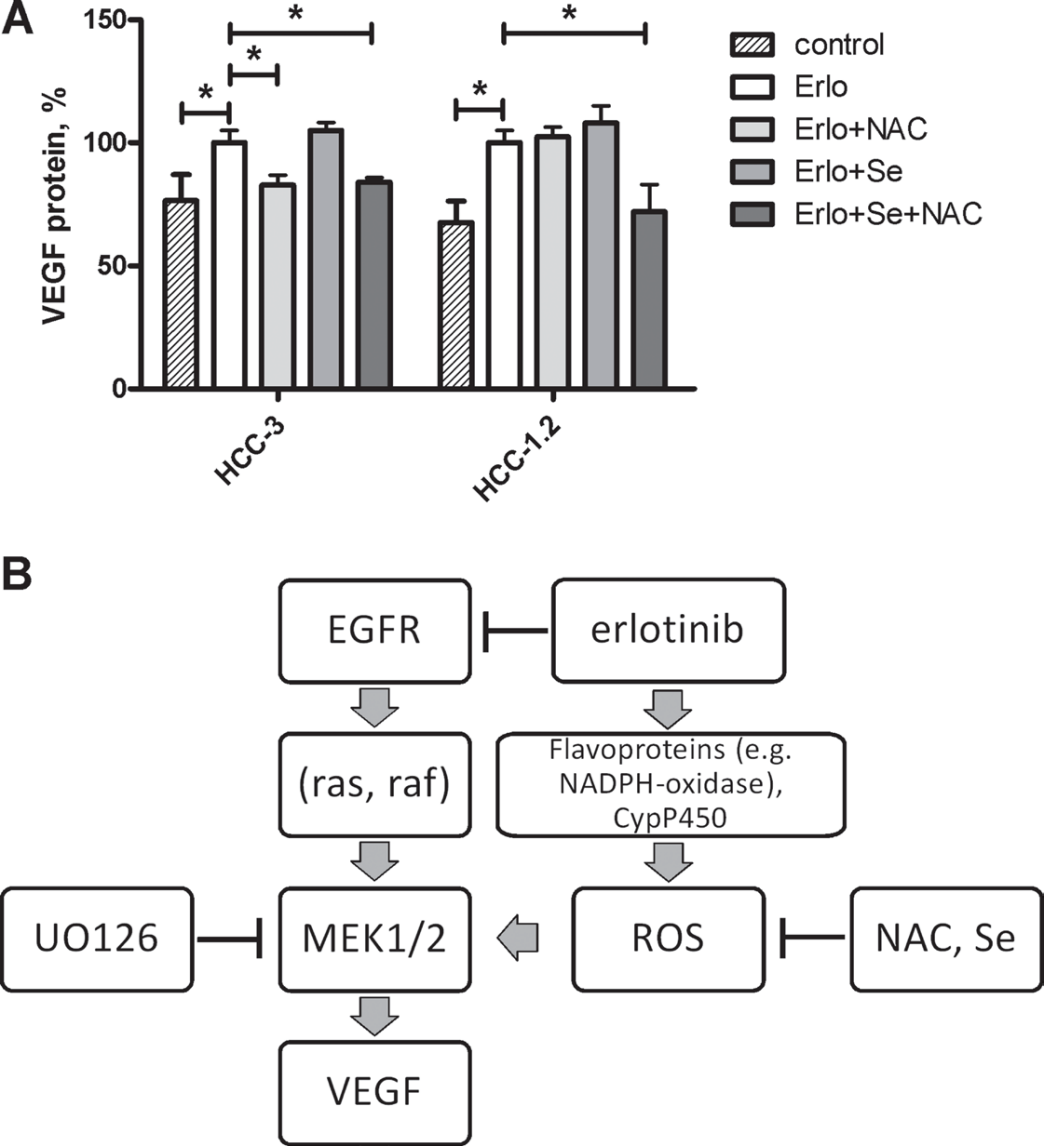
Selenium and N-acetylcystein reduce erlotinib-induced VEGF formation in HCC cells (**A**) 3 mM N-acetylcysteine, 50 nM sodium selenite or both were added to cell medium for 24 h prior to erlotinib treatment (10 μM, 6 h). Supernatants were analysed for VEGF protein formation by means of ELISA. *p < 0.05. (**B**) A mechanism proposed for VEGF induction by erlotinib in human HCC cells.

A combination of selenium with NAC decreased erlotinib-induced VEGF formation in both HCC-1.2 and HCC-3 cells (Figure 6). NAC as a single compound was efficient only in HCC-3 cells. In HCC-1.2 cells, only a combination of NAC with selenium counteracted the erlotinib-induced VEGF formation. Apparently, selenium becomes a limiting factor only if NAC ensures the replenishment of glutathione in HCC-1.2 cells but not in the absence of NAC. In contrast, NAC alone was sufficient to inhibit erlotinib-induced VEGF in HCC-3 cells and combination of NAC with selenium did not further improve the effect.

Thus, exogenous compounds NAC and selenium prevented erlotinib-induced VEGF formation in HCC cells.

## DISCUSSION

Based on microarray gene expression analysis, we identified VEGF as proangiogenic cytokine induced by erlotinib in HCC cells by oxidative stress dependent mechanism (Figure 6B). An increase in VEGF may be rather unfavourable effect in tumour therapy, since VEGF stimulates vessel formation thus leading to nutrient supply and enabling tumour growth. Furthermore, VEGF can accelerate solid tumour growth as an autocrine growth factor, for example in skin cancer [24] as well as in early colon lesions [25]. Our data suggest that the VEGF increase caused by erlotinib may counteract the inhibitory effects of erlotinib on tumour growth thus contributing to therapy resistance, which almost all patients develop over time.

In carcinoma cells, both induction and inhibition of VEGF by erlotinib have been described depending on tumour type. In particular, erlotinib induced VEGF [26] in lung carcinoma cells similar to our data, but decreased VEGF in squamous cell carcinoma [27, 28]. Stimulation of VEGF by erlotinib is not restricted to tumour cells. We have previously shown that erlotinib also increased VEGF release from cultured endothelial cells and serum VEGF in rat HCC [7]. Our present data suggest that tumor cells contribute to systemic increase of VEGF observed earlier.

Increased VEGF is clinically relevant for HCC. Hepatic tumour cells are supposed to be the main contributors to high systemic VEGF levels in HCC patients [29]. In HCC patients, high serum VEGF is associated with tumour recurrence, metastasis and poor survival [30–34].

In contrast to the tumor-promoting role in HCC, VEGF has a dual role in liver fibrosis, being involved both in fibrosis onset [35, 36] as well as in fibrosis resolution [37]. Resolution of experimental liver fibrosis by erlotinib has been described [12], although the possible link to VEGF has not been investigated.

Drug metabolism by CypP450 system is involved in intracellular prooxidant formation [38]. Our data suggest redox-modulation as a mechanism behind VEGF induction by erlotinib. Redox active compounds can activate EGFR in a ligand-independent manner [39]. Contribution of redox imbalance to VEGF formation has been shown for other cell types such as rat FaO hepatoma cells [40] as well as rat glomerular mesangial cells [41]. Similarly, erlotinib enhanced oxidizing reactive oxygen species formation in human FaDu and Cal-27 head and neck squamous cancer cells [42, 43] as well as in lung cancer cells [44]. VEGF increase *in vivo* can result from EGFR blockage not only by erlotinib but also by antibodies, as shown for squamous cell carcinoma [45].

In our study, VEGF upregulation by erlotinib HCC-1.2 cells was clearly dependent on MEK2 which is susceptible to redox interactions. Mechanisms of VEGF upregulation involve redox interactions of MEK/ERK signalling [46]. In Huh7, erlotinib increased only VEGF protein but not mRNA. Therefore, the upregulation mechanisms in Huh7 seem to be different from transcriptional upregulation in HCC-1.2 and HCC-3 and might include e.g. enhanced cap-independent VEGF mRNA translation [47].

Based on our findings, we hypothesize that a combination of the EGFR inhibitor erlotinib with either MEK inhibitor or with N-acetylcysteine/selenium might have beneficial therapeutic effects in humans. Interestingly, a combination of erlotinib with the MEK inhibitor trametinib is currently under clinical investigation (https://clinicaltrials.gov/, study identifiers NCT01192165, NCT01376310).

DCF fluorescence is a two electron oxidation process and is not specific to any particular oxidizing molecules [22]. Application of DPI which inhibits flavoproteins as NADPH-oxidase (NOX), NO-syntase, xanthine oxidase, CypP450 reductase and NADH:ubiquinone oxidoreductase, helped us to identify oxidant production from both flavoprotein-dependent and -independent sources. Our results suggest that both sources contributed to intracellular redox imbalance in erlotinib-treated HCC cells. Indeed, the increased expression of NOX4 has been found to be responsible for superoxide formation under erlotinib treatment in human head and neck cancer (HNSCC) cells [43]. The flavoprotein–independent sources of redox imbalance presumably arise due to hydrogen peroxide and superoxide radical formation during erlotinib metabolism by hepatic cytochrome P450 enzymes, specifically Cyp1A2. Accordingly, ROS were reported to be involved in erlotinib metabolism by cytochrome P450 [48].

Since all four cell lines investigated were originally isolated from human hepatocarcinomas, they represent natural molecular heterogeneity of HCC. However, HCC-1.2 and HCC-3 cell lines established in Austria exhibited higher levels of erlotinib metabolizing enzymes CYP1A2 and CYP3A4 as compared to commercially available Huh7 and SNU398. HCC-1.2 and HCC-3 also showed robust DCF fluorescence increase under erlotinib treatment (Figure 1 and [16]). In contrast, low fluorescence induction by erlotinib was observed in Huh7 and in SNU398 cells with low activity of the main erlotinib metabolising cytochromes P450 (Figure 1 and [49]). Here, we demonstrated the involvement of Cyp1A2 into VEGF induction by erlotnib using specific siRNA.

Both NAC and selenium are exogenous compounds able to reduce intracellular prooxidants in different ways: selenium in form of selenocystein is an constituent of a catalytic centre of hydroperoxide reducing enzymes glutathione peroxidases (GPXs) [50] whereas NAC is a precursor of intracellular glutathione [51] required for hydroperoxide reduction by GPXs . To inhibit erlotinib-induced VEGF formation in HCC cells, we have applied N-acetylcysteine, which is commonly used in the therapy of acute liver failure [52, 53] and reduces liver damage in experimental hepatic fibrosis [54]. Increased radical scavenging by NAC has been proposed as one of the major therapeutic mechanisms [51]. In addition, NAC is a precursor of glutathione formation. Glutathione, in turn, can directly contribute to radical scavenging [55] and is also required for a catalytic activity of selenoenzymes glutathione peroxidases [56].

Under glutathione excess, low selenium can be a limiting factor for efficient hydroperoxide reduction by glutathione peroxidases. Thus, both NAC and selenium contribute to efficient hydroperoxide detoxification. Selenium has been used as enhancer of treatment efficiency by anticancer drugs in models of colon, prostate as well as head and neck tumors [57–61]. To our knowledge, this is the first report proposing the rationale for further animal and human studies on combination of selenium with NAC and as potential enhancers of HCC chemoprevention by erlotinib.

## MATERIALS AND METHODS

### Chemicals

N-acetylcysteine, sodium selenite, and the ERK inhibitor UO126 was purchased from Sigma. Erlotinib (Charge 375904) was a kind gift of Roche Austria (Vienna, Austria). LOOH was synthesized and characterized as described earlier [62]. Briefly, linoleic acid (LH, Sigma) was oxidized for 72 h at room temperature in the dark. The oxidation mixture was dissolved in petroleum ether (boiling range 40–60°C) and extracted four times with water/methanol (1:3 v/v). The obtained aqueous methanol was extracted four times with light petroleum. The methanolic phase was then evaporated under reduced pressure. The concentration of hydroperoxides was calculated using ε_233 nm_ = 25 250 M^−1^ cm^−1^ in ethanol. LOOH stock solution in ethanol was stored in liquid nitrogen.

### Human HCC cell lines

Austrian hepatocarcinoma cell lines HCC-1.2 and HCC-3 have been extensively characterized [16] and were a kind gift of Prof. Bettina Grasl-Kraupp. SNU398 and HUH7 were purchased from ATCC (LGC Standards GmbH, Wesel, Germany). The cell lines were kept under standard tissue culture conditions using RPMI medium containing 10% FCS (Invitrogen, Lofer, Austria) and regularly checked for mycoplasma contaminations. For treatment, cells were seeded into a 6-well plate, grown for 48 h until they reached ∼ 80% confluence. Stock solution of erlotinib was prepared in DMSO. DMSO concentration in the treatment media did not exceed 0.2% vol/vol. 0.2% vol/vol DMSO have been used as a vehicle control and did not affect cell viability.

### Cell viability

Cell viability was determined in triplicates by neutral red assay as described previously [63]. Lactate dehydrogenase (LDH) release into the medium was measured using an enzyme detection kit obtained from Roche Diagnostics according to the manufacturer's instructions. Samples were analysed in duplicates. Cells treated by Triton X100 were used as a positive control.

Apoptosis was assessed by Anexin/7-AAD staining and subsequent FACS analysis as described previously [7].

### Microarray analysis of erlotinib effects on cultured cells

Cultured HCC-1.2 cells were treated for 3 h by 10 μM erlotinib, the concentration within the physiological range measured in humans [18]. mRNA was isolated by a standard Trizol-extraction and treated by DNAse (Quiagen, Venlo, The Netherlands) to eliminate possible DNA impurities. An Affimetrix platform was used to access gene expression patterns. Data were preprocessed using quantile normalization to account for latent batch effects and robust multiarray averaging to summarize probe sets. This combination has been shown to effectively reduce batch effects and to provide good sensitivity for the detection of differentially expressed genes. Three independent experiments were performed. For original microarray data see supplementary files (http://www.ncbi.nlm.nih.gov/geo/query/acc.cgi?acc=GSE67545). Comparative enrichment analysis was performed by Genego platform (Thomson Reuters). The threshold was set to 1.15. Only the genes regulated at *p* < 0.001 were considered.

### DHFC staining

DHFC is a redox sensitive probe which is cell permeable and udergo two-electron oxidation resulting in formation of a fluorescent dichlorofluorescein (DCF) [22]. Staining procedure has been described in our earlier work [19]. Briefly, cells were grown until 60–80% confluence, harvested, mixed with 20 μM 2′,7′-dichlorofluorescin diacetate (DHFC) and exposed to a 10μM erlotinib for 2 h at 37°C in Hanks’ balanced saline solution (HBSS), containing Ca^2+^, Mg^2+^ and 1% FCS. A Coulter Cell Lab Quanta SC flow cytometer with Cell Lab Quanta Analysis software was used to quantify DCF formation.

### Application of linoleic acid hydroperoxides (LOOH)

Immediately before use, LOOH-stocks were diluted into serum free medium containing 1mg/mL fatty acid free BSA and dispersed by sonication three times for 5 s. The final concentration of ethanol in the medium did not exceed 0.1%.

### Isolation of mRNA and real time reverse transcriptase (RT)-PCR

mRNA was isolated according to a standard Trizol-extraction protocol (Invitrogen, Austria). The purity and quantity of the mRNA was determined using gel electrophoresis and photometry. cDNA synthesis was performed on 2 μg of the total mRNA by High Capacity cDNA Reverse Transcription Kit (Applied Biosystems Inc., Foster City, USA).

To analyse VEGF mRNA expression by real time RT-PCR, Taqman System and primer Hs00173626_m1 (Applied Biosystems Inc., Foster City, USA) were applied. The ΔΔCt method was used for calculations.

### Interventions by siRNA

We have used Ambion Silencer Select siRNA to transfect HCC-1.2 and HCC-3 cells. The cells were seeded into the 6-well plates and grown for 24h in a full medium. For transfection, Lipofectamine RNAiMAX (Invitrogen) and OptiMem medium were used according to the manufacturers’ instructions. siRNA concentration was 5 nM in test, the transfection time was 24 h for MEK1 and MEK2 siRNA and 48 h for Cyp1A2 and Cyp3A4 siRNA. Following siRNAs have been used (all from Ambion): MEK1 s11168; MEK2 s11171; CYP1A2 s3304; CYP3A4 s3846; negative control No. 4390846. To prove the efficiency of silencing, mRNAs were analysed by real time RT-PCR using the following primers: MEK1 Hs00983247_g1; MEK2 Hs 00829210_s1. GAPDH was used as a house keeping gene.

### VEGF protein measurements

Human VEGF was determined by Quantikine ELISA Kit (R&D Systems, Abingdon, UK) used according to the manufacturers’ instructions.

### Western blotting

Cells were washed twice with PBS, and harvested in ice-cold lysis buffer containing 50 mM Tris-HCl (pH 7.4), 0.1 mM EGTA, 0.1 mM EDTA, 2 mM leupeptin, 1 mM phenylmethylsulfonyl fluoride, 1% Nonidet P40, 0.1% sodium dodecyl sulfate, and 0.1% deoxycholate. Samples were then centrifuged for 30 minutes at 10,000 g. Protein concentration in supernatant was measured using a BCA colorimetric assay (Thermoscientific Rockford, IL, USA). Protein lysates (100 μg) were separated by SDS-PAGE and transferred to nitrocellulose membranes. Membranes were blocked with 5% non-fat dry milk incubation buffer and exposed to respective primary antibodies. Following antibodies were used: pERK (Santa Cruz Biotechnology Inc., Santa Cruz, USA) and total ERK. A secondary peroxidase-linked antibody was used for chemiluminescent detection. Loading accuracy was evaluated by membrane rehybridization with monoclonal antibodies against GAPDH or β-actin. Quantification was carried out by Image Quant 7.0 Software.

### Statistics

All experiments were performed at least 3 times as independent biological replicates. If not indicated otherwise, data are expressed as mean + SEM, and statistical differences were determined using one-way ANOVA with significance considered at *p* < 0.05.

## CONCLUSIONS

In summary, the findings of our study uncovered the molecular mechanisms of VEGF induction by the EGFR inhibitor erlotinib in HCC cells. Counteracting such mechanism by MEK2 inhibitors, N-acetylcysteine and selenium may improve the therapeutic efficacy of erlotinib. Redox imbalance and VEGF increase resulting from erlotinib metabolism should be taken into account for the development of novel therapeutic strategies targeting therapy and chemoprevention of liver cancer.

## SUPPLEMENTARY MATERIALS FIGURES AND TABLES


