# Supplementary material for: Oxidative stress mediates an increased formation of vascular endothelial growth factor in human hepatocarcinoma cells exposed to erlotinib
Source: Oncotarget. 2017 Jul 6;8(34):57109–20. doi: 10.18632/oncotarget.19055 (PMC5593629; doi:10.18632/oncotarget.19055)
Supplement: Supplementary file 1 [file oncotarget-08-57109-s001.pdf]

Oxidative stress mediates an increased formation of vascular endothelial growth factor in human hepatocarcinoma cells exposed to erlotinib

SUPPLEMENTARY MATERIALS

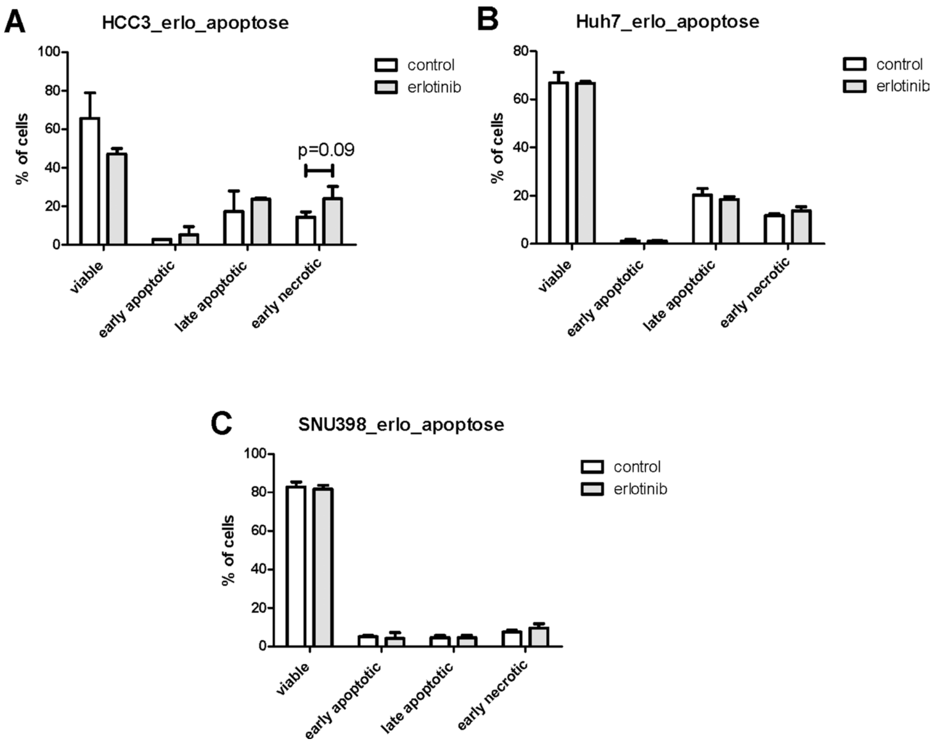

Supplementary Figure 1: Measurements of apoptosis in HCC cell lines incubated with erlotinib. HCC-3, Huh7 and SNU398 cells were treated eother by 5 µM erlotinib (erlotinib) or by solvent control (control) for 72 h in full medium and analysed for apoptosis by 7-AAD/Annexin staining with subsequent FACS analysis as describes earlier [7]. Three independent experiments have been performed.

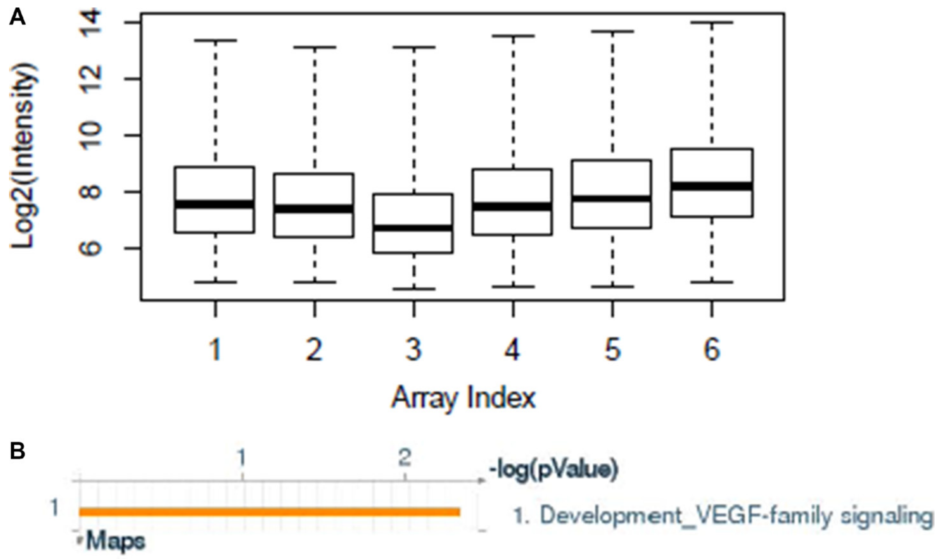

Supplementary Figure 2: Microarray analysis of HCC-1.2 cells treated ba erlotinib. HCC-1.2 cells were treated by 10 µM Erlotinib for 3 hours and microarray gene expression analysis has been conducted. (**A**) Boxplots of the preprocessed expression values on each chip. Array index indicates the following chips: repeat 1:1-solvent control, 2 - erlotinib ; repeat 2:3- solvent control, 4- erlotinib; repeat 3: 5- solvent control, 6 - erlotinib. (**B**) Results of sorting is done for the 'Statistically significant Maps" performed using comparative enrichment analysis by Genego plattform as described in Materials and Methods.

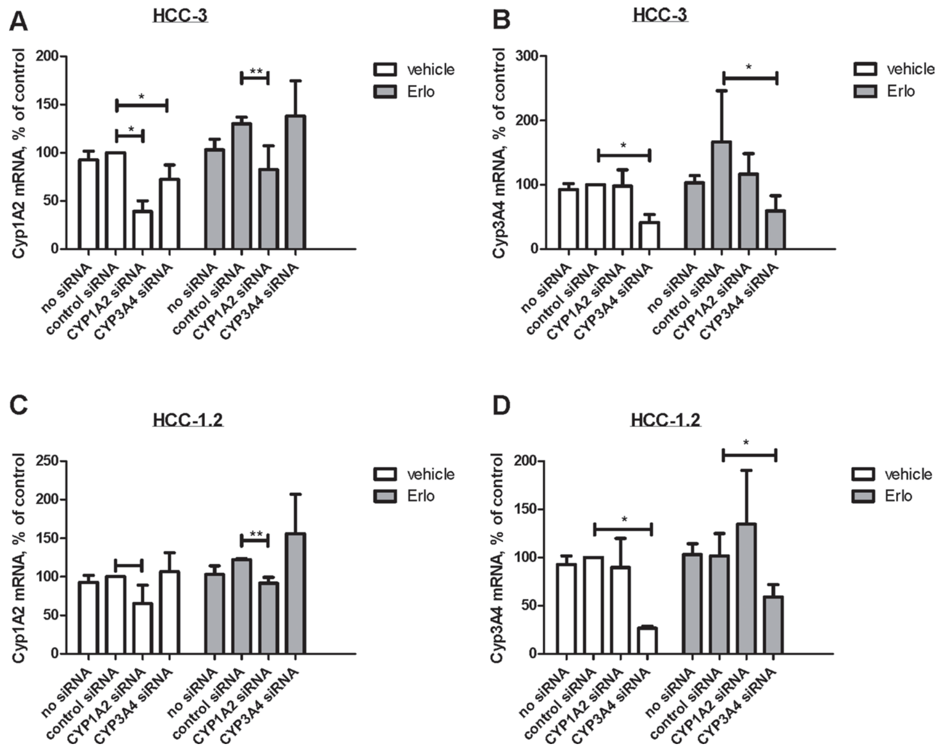

Supplementary Figure 3: The efficiency and cross-interaction of siRNAs against CYP1A2 and CYP3A4 in HCC-3 and HCC-1.2 cells. Transfection has been performed using Lipofectamin and 5nM siRNA for 48 hours as described in Materials and Methods. Afterwords, the cells were treated by 10 µM erlotinib (Erlo) or by solvent control (vehicle) for 6 hours and CYP3A4 as well as CYP1A2 mRNA has been investigated by real-time RT-PCR using GAPDH as a houskeeping gene. The results for CYP1A2 (**A**) and CYP3A4 (**B**) in HCC-3 as well as for CYP1A2 (**C**) and CYP3A4 (**D**) mRNA in HCC-1.2 cells are presented. The results of $n = 2–3$ independent experiments are summerized. *$p < 0.05$; **$p < 0.01$.

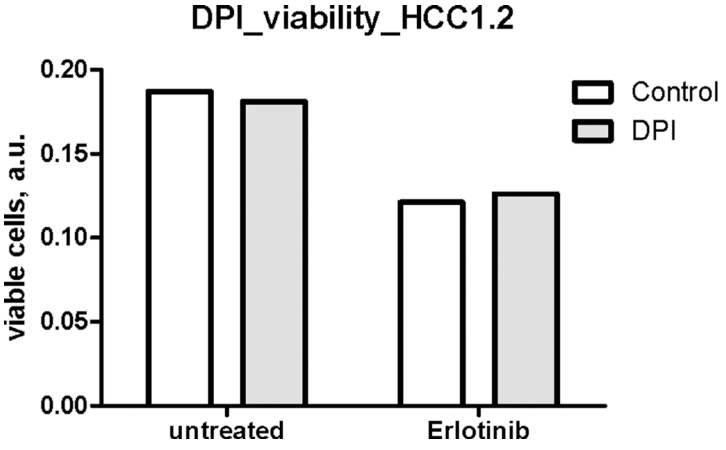

Supplementary Figure 4: Impact of diphenylene iodonium (DPI) on viability of HCC-1.2 cells exposed to erlotinib. Untreated cells or cells exposed to 10 µM erlotinib (white bars) were additionaly incubated with 15 µM DPI (grey bars) in 6-well plates for 4 hours and viable cells were quantified by neutral red assay as described in Materials and Methods. Means of $n = 3$ experiments are shown.

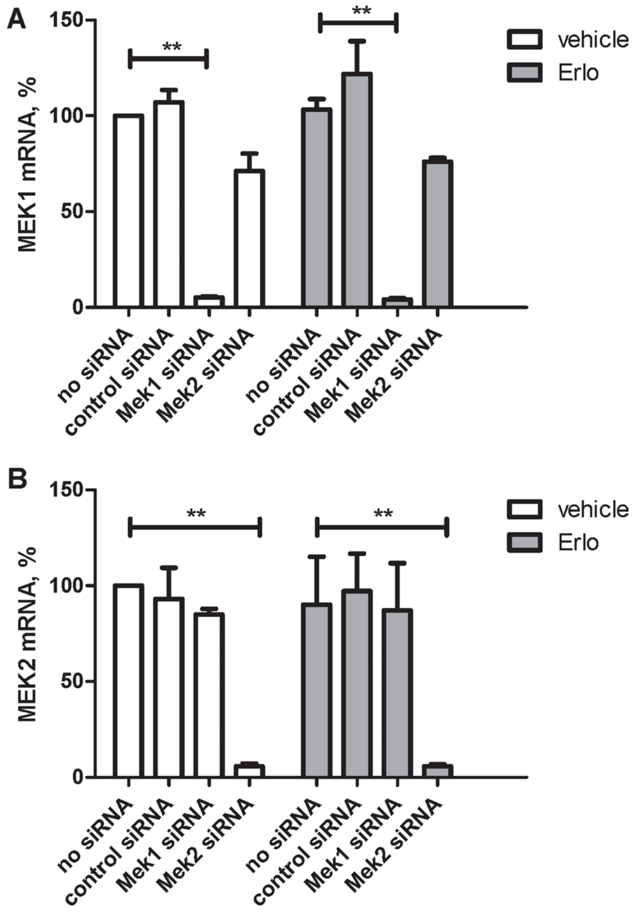

Supplementary Figure 5: The efficiency and cross-interaction of siRNAs against Mek1 and Mek2 in HCC-1.2 cells. Transfection has been performed using Lipofectamin and 5 nM siRNA for 48 hours as described in Materials and Methods. Afterwords, the cells were treated by 10 µM erlotinib (Erlo) or by solvent control (vehicle) for 6 hours and Mek1 as well as Mek2 mRNA has been investigated by real-time RT-PCR using GAPDH as a houskeeping gene. The results for Mek1 (**A**) and Mek2 (**B**) are presented. The results of $n = 2–3$ independent experiments are summerized. $^{**}p < 0.01$.

Supplementary Table 1: The most strongly regulated genes in HCC-1.2 cells treated by erlotinib

ttest	mea (CO)	mean (Erlo)	FC (Erlo/CO)	Gene Symbol	Gene Description
0,001	39,0320387	32,4287725	0,83082446	F13B	coagulation factor XIII, B polypeptide
0,000	63,8194249	53,1761499	0,83322828	EDN3	endothelin 3
0,001	17,1065221	14,720514	0,86052056	GTSCR1	Gilles de la Tourette syndrome chromosome region, candidate 1
0,001	88,4212031	77,1995278	0,87308841	ZNF211	zinc finger protein 211
0,001	106,801894	93,9067989	0,87926155	COPG2	coatomer protein complex, subunit gamma 2
0,001	77,7415373	68,4833162	0,88091024	IGSF9B	immunoglobulin superfamily, member 9B
0,000	205,982458	185,431679	0,90023044	KLHL18	kelch-like 18 (Drosophila)
0,001	1084,69692	1119,6589	1,03223203	MRPL36	mitochondrial ribosomal protein L36
0,001	11,3257955	11,92582	1,05297857	KLRC1	killer cell lectin-like receptor subfamily C, member 1
0,000	246,506858	263,016455	1,06697419	TNFRSF13B	tumor necrosis factor receptor superfamily, member 13B
0,000	324,212681	347,056186	1,0704584	STK25	serine/threonine kinase 25 (STE20 homolog, yeast)
0,001	98,5250835	106,330288	1,07922049	NEIL2	nei like 2 (E. coli)
0,000	141,06547	152,488881	1,08097949	KIAA0247	KIAA0247
0,001	78,1769503	84,5127464	1,0810443	WNT3A	wingless-type MMTV integration site family, member 3A
0,000	72,7960271	79,2239391	1,08830031	TRH	thyrotropin-releasing hormone
0,001	105,288794	115,187883	1,09401845	POU3F2	POU class 3 homeobox 2
0,000	191,391315	211,028107	1,10260023	KANK1	KN motif and ankyrin repeat domains 1
0,001	10,4812017	11,7439492	1,12047737	WDR17	WD repeat domain 17
0,001	745,099788	838,160016	1,12489633	PCYT2	phosphate cytidylyltransferase 2, ethanolamine
0,000	114,664869	131,228313	1,1444509	NACAD	NAC alpha domain containing
0,000	96,3626342	110,867036	1,15051894	KCNA10	potassium voltage-gated channel, shaker-related subfamily, member 10
0,001	105,67702	122,084687	1,15526239	VEGF	vascular endothelial growth factor
0,001	427,361563	495,757141	1,16004148	PEX26	peroxisomal biogenesis factor 26
0,001	30,4813983	35,8685606	1,17673606	CD200	CD200 molecule
0,001	140,90469	168,433105	1,19536905	RAB15	RAB15, member RAS onocogene family
0,000	28,2111446	34,698071	1,22994198	LYZL6	lysozyme-like 6
0,001	583,08707	720,187069	1,23512783	TIPARP	TCDD-inducible poly(ADP-ribose) polymerase
0,001	1354,406	1688,26599	1,2464992	INHBE	inhibin, beta E

Supplementary Table 2: The results of gene set enrichment approach of microarray gene expression analysis of erlotinib-treated HCC-1.2 cells

	holm	alias
04115	0.01	p53 signaling pathway
00250	0.02	Alanine, aspartate and glutamate metabolism
04060	0.04	Cytokine-cytokine receptor interaction
04742	0.06	Taste transduction
00534	0.11	Glycosaminoglycan biosynthesis - heparan sulfate
00830	0.17	Retinol metabolism
05222	0.20	Small cell lung cancer
00270	0.27	Cysteine and methionine metabolism
04722	0.28	Neurotrophin signaling pathway
00533	0.33	Glycosaminoglycan biosynthesis - keratan sulfate

The data were analyzed using a gene set enrichment approach. To this end for each of a collection of predefined gene sets is the combined null hypotheses that none of the included genes is differentially expressed is tested using the global test. This test has improved sensitivity in situations where many genes collectively demonstrate a coordinated effect.
